# Macular pigment optical density of hyperopic anisometropic amblyopic patients measured by fundus reflectometry

**DOI:** 10.3389/fmed.2022.991423

**Published:** 2022-10-11

**Authors:** Chenxiao Wang, Jinjin Yu, Mengmeng Pan, Xiuhong Ye, E. Song

**Affiliations:** ^1^Department of Ophthalmology, The Second Affiliated Hospital of Soochow University, Suzhou, China; ^2^Department of Ophthalmology, Lixiang Eye Hospital of Soochow University, Suzhou, China; ^3^Eye Hospital and School of Ophthalmology and Optometry, Wenzhou Medical University, Wenzhou, China

**Keywords:** macular pigment optical density, macular foveal thickness, fundus reflectometry, hyperopic anisometropic amblyopia, children

## Abstract

**Purpose:**

Hyperopic anisometropia is a major cause of amblyopia and may be associated with macular pigment optical density (MPOD) reduction. To explore whether the MPOD changes in hyperopic anisometropic amblyopia, we measured the MPOD using fundus reflectometry in eyes with hyperopic anisometropic amblyopia and normal vision.

**Methods:**

This was a cross-sectional study conducted from January 2017 to June 2017. Forty subjects (25 males and 15 females) between the ages of 6 and 10 years were recruited. The subjects' eyes were divided into two groups: amblyopic eyes (best-corrected visual acuity (BCVA) not more than 20/25 or BCVA of two eyes differing by two or more lines) and fellow eyes. All enrolled subjects underwent a comprehensive ophthalmic examination, including extraocular motility assessment, cover-uncover testing, and refractive error (noncycloplegic), BCVA, axial length (AL), macular foveal thickness (MFT) and MPOD (Visucam^®^ 200, Carl Zeiss Meditec AG, Germany).

**Results:**

The MPOD of amblyopic and fellow eyes was 0.12 ± 0.03 log units and 0.13 ± 0.04 log units, respectively, with a significant difference (*P* = 0.026). The MFT of amblyopic and fellow eyes was 241.28 ± 13.95 and 237.13 ± 16.02 μm, respectively, revealing that the MFT was significantly higher in amblyopic eyes than in fellow eyes (*P* = 0.028). Conversely, there was no correlation between the MPOD and MFT in the two groups.

**Conclusions:**

This study is the first to report that the MPOD is decreased in hyperopic anisometropic amblyopia. In this study, no correlation between the MPOD and MFT was found. In the future, factors that induce a decrease in the MPOD in eyes with hyperopic anisometropic amblyopia should be explored in a large-sample study with follow-up observation.

## Introduction

Amblyopia refers to abnormal visual experience of strabismus, refractive error, anisometropia and form deprivation during the critical period of visual development. In amblyopia, the visual stimulus that enters the eye is not sufficiently clear, thus resulting in a below-normal best-corrected visual acuity (BCVA) in one or both eyes, with no organic eye lesion (1). The prevalence rates of amblyopia in children vary from 1 to 5% (2, 3). To date, some studies have pointed out that there are pathological changes in the morphology and function of the visual pathway and visual center in amblyopic patients. The decrease in visual information input leads to the development of a neuron disorder in the visual cortex and atrophy of lateral geniculate nucleus cells, which leads to a decrease in the visual system's response to visual stimulation (4–7). Furthermore, with recent developments in fundus imaging technology, studies have observed abnormal retinal morphology, including changes to the retinal ganglion cells, the retinal nerve fiber layer (RNFL), and the optic nerve, in amblyopic patients (8, 9). However, most of these studies have focused on the thickness of the retina or the retinal structure, and the density and function of cone cells in the macular region have not been fully studied. Bruce et al. demonstrated that there are differences in the foveal structure between amblyopes and nonamblyopes; however, the functional significance and underlying mechanism are uncertain (10). At present, the standard treatment for hyperopic anisometropic amblyopia is occlusion of the dominant eye. However, due to cosmetic dissatisfaction and the impact on daily activities, compliance with this treatment is poor. Thus, many other treatments have emerged, such as levodopa treatment (11) and excimer laser refractive surgery (12). Since the efficiency of these treatments remains unclear, the early detection of hyperopic anisometropic amblyopia and undertaking of mechanistic research are important.

Macular pigment (MP) is mainly composed of lutein (L), zeaxanthin (Z) and meso-zeaxanthin, which are unevenly distributed throughout the retina (13). In horizontal sections, the concentration was the highest in the macular fovea and decreased sharply toward the periphery of the fovea (14). In longitudinal sections, MP was concentrated in the axons of cone cells, outer segment and inner layer of rod cells (15, 16). The development of MP is synchronized with changes in the arrangement direction and density of neurons around optic cone cells in the macula (17). Therefore, the evaluation of MP plays an important role, and the value in its detection lies in exploring the mechanism of and relationship between amblyopia and changes in macular retinal morphology.

The macular pigment optical density (MPOD) is a measure of the light absorption characteristics of MP (18, 19). At present, techniques for measuring the MPOD *in vivo* are divided into psychophysical methods and objective methods. The former includes heterochromic flicker photometry and minimum motion photometry (20–22), and the latter includes fundus reflectometry, fundus autofluorescence, resonance Raman spectroscopy, and visual-evoked potentials (23, 24). Fundus reflectometry, which can be used to measure the MPOD, is the quantitative assessment of the amount of light reflected from the fundus. There are two methods that predominate. The first utilizes two wavelengths (one absorbed by MP and one not) to compare light reflected from the fovea with light reflected from an eccentric retinal area. The second involves single-wavelength reflectometry to analyze the spectrum of light reflected from a spot on the retina (24). Some researchers have employed this device to measure the MPOD of patients with diabetes and age-related macular degeneration, reporting decreased MPOD values (25–28). In this study, we used fundus reflectometry to study the MPOD in eyes with hyperopic anisometropic amblyopia and normal vision to explore whether the MPOD is altered in hyperopic anisometropic amblyopia, to further analyze the pathogenesis of amblyopia and identify possible auxiliary means for the treatment of amblyopia.

## Materials and methods

### Subjects

Forty subjects between the ages of 6 and 10 years were recruited from Wenzhou Medical University between January 2017 and June 2017. This project was approved by the Ethics Committee of Wenzhou Medical University, and written consent was obtained from all subjects and their guardians after they were informed about the benefits, risks, and possible adverse consequences of the procedures. The research was performed in compliance with the tenets of the Declaration of Helsinki.

All enrolled subjects underwent a comprehensive ophthalmic examination, including extraocular motility assessment, cover-uncover testing, and refractive error (noncycloplegic), best-corrected visual acuity (BCVA), axial length (AL) (Lenstar 900^®^, Haag-Streit AG, Switzerland), macular foveal thickness (MFT) (iVue^®^ 100, Optovue, America) and MPOD (Visucam^®^ 200, Carl Zeiss Meditec AG, Germany). Subjects with a BCVA in one eye of not more than 20/25 and in the other eye of not < 20/20 or for whom the BCVA of both eyes differed by two or more lines and the spherical equivalent (SE, defined as the spherical power plus half of the minus cylinder power) was more than +1.00 D were enrolled in the amblyopic group. Their other normal eyes were included in the fellow group. The exclusion criteria included the following: patients with strabismus amblyopia or form deprivation amblyopia caused by a congenital cataract; patients with an ocular pathology, trauma or a history of surgery; patients with a systemic disease affecting eye health, such as diabetes and hypertension; and patients who were insufficiently cooperative to be examined.

### MPOD and MFT measurement

The Visucam^®^ 200 (Visucam^®^ 200, Carl Zeiss Meditec AG, Germany) is a type of fundus digital camera that not only captures normal stereo images and processes the fundus but also innovatively measures the MPOD by fundus reflectance. The Visucam^®^ 200 is an objective tool for measuring the MPOD in a simple, fast and noninvasive manner. The high repeatability and reproducibility of the Visucam^®^ 200 in MPOD measurement have already been proven (29, 30). The MPOD module for the Visucam^®^ 200 uses the reflectance of the narrow-band wavelength (480 to 500 nm) to determine the MPOD and its spatial distribution (30). Four MPOD parameters are automatically calculated by the software: the area (area of 3.5° eccentricity), volume (sum of all ODs within the measurement area), max OD (the point where maximum pigment density is found in the macula), and mean OD (the mean MP density obtained over the measurement area). Due to the product's patent, the specific digital model for processing fundus images has not been disclosed. Therefore, the parameter selected in this study was the mean OD.

The retina was imaged using the Optovue RTVue XR Avanti (Optovue, Inc., Fremont, CA, USA) optical coherence tomography (OCT) system. Eighteen consecutive transverse B-scans of the fundus were captured by 8-mm radial line scans passing through the fovea. Good sets of scans with a signal strength index of more than 40 were selected for further analysis. The MFT from the internal limiting membrane (ILM) to the retinal pigment epithelium (RPE) within an area 1 mm in diameter was measured and averaged (31).

### Statistical analyses

Statistical analyses were performed using SPSS (version 22.0; SPSS, Inc., Chicago, IL, USA). The Shapiro–Wilk test was used to evaluate the normality of the data distribution. Comparisons were performed using paired *t*-tests. Correlations were assessed using partial correlation analysis after adjusting for the AL. *P*-values < 0.05 were considered statistically significant.

## Results

### Patient characteristics

Table 1 presents demographic data for all the subjects. Among the amblyopic patients (25 males and 15 females), the mean age was 9.57 ± 2.08 years. The SE of the amblyopic and fellow eyes was 5.23 ± 1.64 D and 1.13 ± 1.15 D, respectively, with a significant difference (*P* < 0.001). There was also a significant difference in the AL between the two groups (*P* < 0.001). The AL was shorter in the amblyopic eyes (21.69 ± 0.84 mm) than in the fellow eyes (23.09 ± 0.77 mm).

**Table 1:** Characteristics of amblyopic eyes and fellow eyes.

**Group**	**Amblyopic eyes (*n =* 40)**	**Fellow eyes (*n =* 40)**	***t***	***P*-value**
Sex (male/female)	25/15	25/15		
SE (D)	5.23 ± 1.64	1.13 ± 1.15	15.378	< 0.001
AL (mm)	21.69 ± 0.84	23.09 ± 0.77	−14.933	< 0.001
LogMAR	0.57 ± 0.26	0.01 ± 0.02	13.74	< 0.001
Mean MPOD (log units)	0.12 ± 0.03	0.13 ± 0.04	−2.314	0.026
MFT (μm)	241.28 ± 13.95	237.13 ± 16.02	2.282	0.028

SE, spherical equivalent; AL, axial length; MPOD, macular pigment optical density; MFT, macular foveal thickness.

### Comparison of MPOD and MFT in amblyopic and fellow eyes

Table 1 shows the MPOD and MFT of the amblyopic and fellow eyes. The MPOD in the amblyopic and fellow groups was 0.12 ± 0.03 log units and 0.13 ± 0.04 log units, respectively, with a significant difference (*P* = 0.026). Moreover, the MPOD was significantly lower in the amblyopic eyes than in the fellow eyes. The MFT of the amblyopic and fellow eyes was 241.28 ± 13.95 and 237.13 ± 16.02 μm, respectively, with a significant difference (*P* = 0.028); the MFT was significantly greater in the amblyopic eyes than in the fellow eyes.

### Correlations among the MPOD, MFT, SE, and BCVA

**Figure 1:**
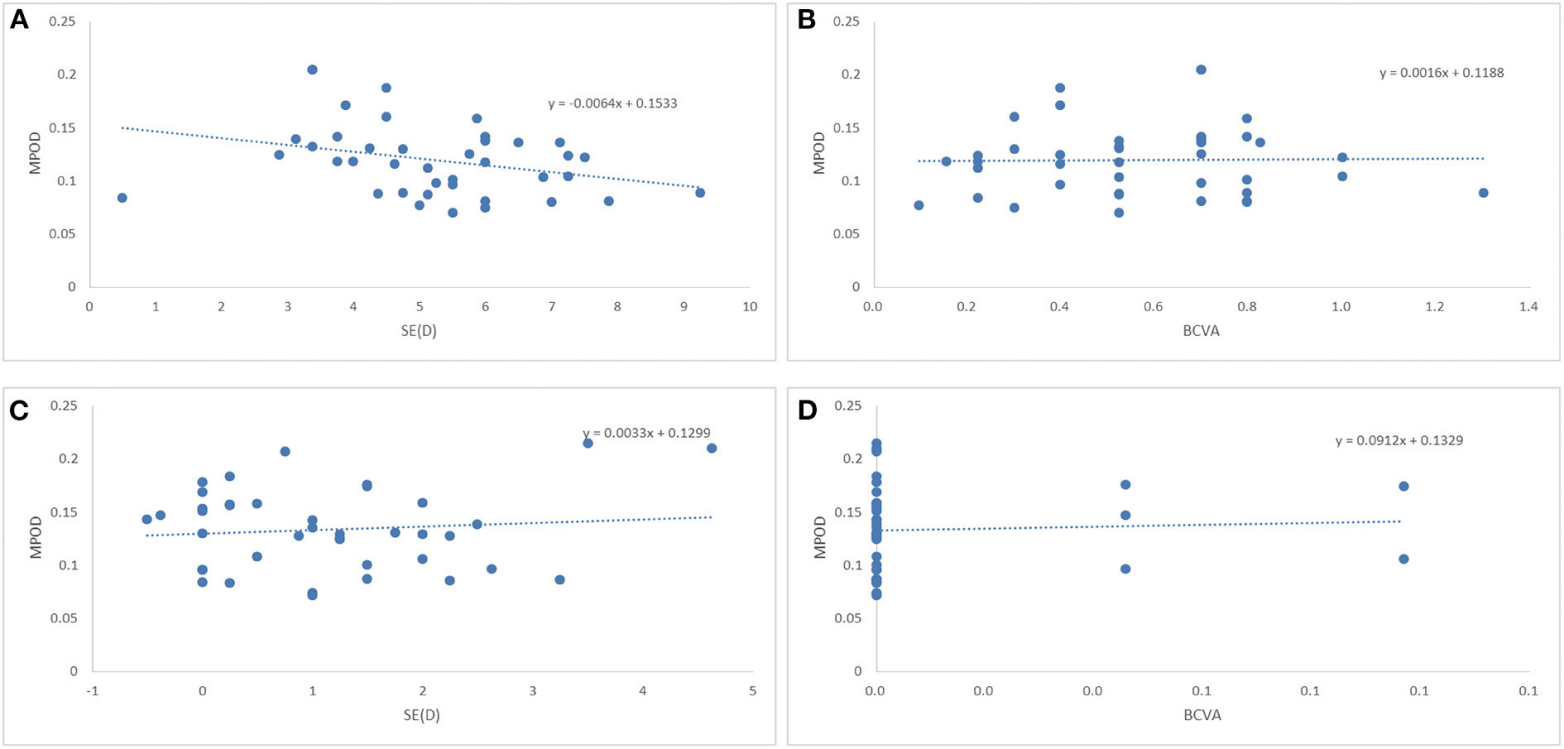
Correlations among the macular pigment optical density (MPOD), equivalent spherical power (SE) and best-corrected visual acuity (BCVA) in amblyopic and fellow eyes. **(A)** Comparison of the MPOD and SE in amblyopic eyes. **(B)** Comparison of the MPOD and BCVA in amblyopic eyes. **(C)** Comparison of the MPOD and SE in fellow eyes. **(D)** Comparison of the MPOD and BCVA in fellow eyes.

Partial correlation analysis showed that there was no correlation of the MPOD or MFT with the SE or BCVA in the two groups (Tables 2, 3 and Figure 1). Moreover, there was no correlation between the MPOD and MFT in the two groups.

**Table 2:** Correlation of the MPOD and MFT with the SE and BCVA in the amblyopic group.

		**SE**	**LogMAR**	**MPOD**
MPOD	Correlation coefficient	−0.309	0.012	–
	*P*	0.052	0.941	–
MFT	Correlation coefficient	0.015	0.114	−0.007
	*P*	0.926	0.482	0.965

MPOD, macular pigment optical density; MFT, macular foveal thickness; SE, equivalent spherical power; BCVA, best-corrected visual acuity.

**Table 3:** Correlation of the MPOD and MFT with the SE and BCVA in the fellow group.

		**SE**	**LogMAR**	**MPOD**
MPOD	Correlation coefficient	0.098	0.057	
	*P*	0.546	0.725	
MFT	Correlation coefficient	0.045	0.286	0.213
	*P*	0.783	0.073	0.187

MPOD, macular pigment optical density; MFT, macular foveal thickness; SE, equivalent spherical power; BCVA, best-corrected visual acuity.

## Discussion

The correlation between amblyopia and the MPOD is rarely reported in the literature. In this study, we first evaluated the MPOD in children with hyperopic anisometropic amblyopia. We found that the MPOD of hyperopic anisometropic amblyopic eyes was lower than that of fellow eyes and that the MFT of amblyopic eyes was thicker than that of fellow eyes, with significant differences. However, there was no correlation between the MPOD and MFT in the two groups.

In the current study, we found that the MFT was increased in amblyopic eyes compared with fellow eyes. Although some researchers believe that the macular thickness of eyes with monocular amblyopia (anisometropia, strabismus, or mixed) is not different from that of the fellow eyes, most researchers believe that the foveal thickness of eyes with anisometropic amblyopia is thicker than that of the fellow eyes (9, 32, 33). Andalib et al. used OCT to measure the macular thickness of 25 anisometropic amblyopic patients aged 6–18 years and found that the MFT (222.6 ± 47.8 μm) of the amblyopic eye was thicker than that of the contralateral eye (205.6 ± 33.3 μm) (34). Al-Haddad et al. also used high-definition spectral-domain OCT to examine 31 patients with anisometropia and found that the MFT of the amblyopic eye (273.8 μm) was thicker than that of the contralateral eye (257.9 μm) (4). Bandyopadhyay et al. (35) and Huynh et al. (6) also obtained the same conclusions. This finding indicates that the anisometropic amblyopic process might involve the macula.

With respect to the MPOD, we found that the MPOD of hyperopic anisometropic amblyopic eyes was lower than that of fellow eyes. Anatomically, MP is concentrated in the axons of cone cells, the outer segment and the inner layer of rod cells. Liao et al. found that the cone density expressed in angular density units in amblyopic eyes was lower than that in fellow eyes and normal controls, which supports our conclusion (9). In addition, molecular biology studies have indicated that L and Z may reflect density changes in rod cells and cone cells (36). At present, there is insufficient clinical evidence to prove the influence of L and Z intake and MPOD on the visual system. However, animal experiments have proven that a lack of L and Z intake after birth can lead to significant abnormal changes in retinal structure (37–39). In 2005, Barker et al. showed that supplementation with L and Z could partly repair retinal damage (39).

With the continuous reduction in cone diameter and the increase in cell density after birth, cone cells gradually occupy the dominant position in the fovea, the MP density gradually increases to near the adult level, and visual function improves rapidly after birth. If the abnormal visual experience deprives the macular region of the opportunity to receive normal visual stimulation after birth, the impact on the increase in cone cell density and the thinning of the retina will simultaneously affect the development of MP and cause a relative decrease in MP density (40).

Therefore, there are several reasons for the decrease in MPOD in amblyopic eyes. One is that the process of foveal formation is blocked, the foveal thickness is increased (4, 34, 35), and the relative distribution volume of MP is increased. Another possibility is that the amount of trans-zeaxanthin transformed in the axons of the cone cells is reduced when the cone cells are arranged in a disorderly manner. In addition, it has been suggested that MPOD levels may be genetically influenced (41). Nonetheless, studies of MPOD in monozygotic twins have shown that MP levels are not entirely genetic (42). At present, there is no research on the level of MP production in amblyopia worldwide; thus, further research and exploration are needed.

Regardless of whether measured in the amblyopic eye or fellow eye, the MPOD showed no correlation with the MFT. It is known that MP accumulates in the outer plexiform layer of the retina, and changes in the MFT in amblyopic eyes do not accurately represent the thickness of this structure. To accurately explore the relationship between the density of MP and the thickness of each layer of the retina, in follow-up research, self-designed high-resolution OCT will be used to measure the thickness of each layer of the retina.

The fundus reflectometry approach used in this study is a simple noninvasive technique for measurement of the MPOD, which can be used for the assessment of fovea. As an objective method, it requires minimal patient effort and has a quick measurement time. The most important aspect is the good reliability among instruments (43, 44). Additionally, it is suitable for many subject populations, including children (24). Therefore, fundus reflectometry can be considered a good choice among various methods. To the best of our knowledge, this study is the first to report the use of fundus reflectometry to measure the MPOD in amblyopic patients.

One limitation of this study is that the sample size was relatively small, which may be the main reason why the MPOD, SE and BCVA were not found to be associated. In the next study, we will use a larger sample. In addition, we will include normal hyperopic individuals to determine whether there are statistically significant differences when comparing hyperopic anisometropic amblyopic eyes with normal hyperopic eyes. This was also a cross-sectional study. In the future, longitudinal comparisons of individuals will be used to further explore the correlation between hyperopic anisometropic amblyopia and MPOD. In addition, this study did not include information regarding the use of L or Z supplements or dietary patterns. Therefore, the possible influence of the above factors cannot be excluded.

This study is the first to report that the MPOD decreases in hyperopic anisometropic amblyopia. No correlation between the MPOD and SE, BCVA or MFT was found. The factors inducing the decrease in MPOD in eyes with hyperopic anisometropic amblyopia need to be explored in future studies with a large sample and follow-up design.

## Data availability statement

The original contributions presented in the study are included in the article/supplementary materials, further inquiries can be directed to the corresponding author/s.

## Ethics statement

The studies involving human participants were reviewed and approved by the Ethics Committee of Wenzhou Medical University. Written informed consent to participate in this study was provided by the participants' legal guardian/next of kin.

## Author contributions

CW participated in the conception and design of the research study. CW and ES wrote and revised the manuscript. All the authors contributed to the collection of the data and the statistical analyses of the data.

## Funding

This work was supported in part by the Jiangsu Provincial Natural Science Foundation Project (SBK2019022051), Jiangsu Distinguished Medical Experts Program (2016), and Suzhou Science and Technology Bureau (SYS2018004). These sponsors or funding organizations played no role in the design or conduct of this research.

## Conflict of interest

The authors declare that the research was conducted in the absence of any commercial or financial relationships that could be construed as a potential conflict of interest.

## Publisher's note

All claims expressed in this article are solely those of the authors and do not necessarily represent those of their affiliated organizations, or those of the publisher, the editors and the reviewers. Any product that may be evaluated in this article, or claim that may be made by its manufacturer, is not guaranteed or endorsed by the publisher.
